# Transluminal cholangioscopy via hepaticogastrostomy for a refractory bilioenteric anastomotic stricture using a novel slim cholangioscope

**DOI:** 10.1055/a-2987-9037

**Published:** 2026-11-03

**Authors:** Wei Zhang, Rabii Ajana, GuanHua Chan, Senlin Hou, Lichao Zhang

**Affiliations:** 1 Biliopancreatic Endoscopic Surgery Department 71213 Second Hospital of Hebei Medical UniversityShiJiaZhuangHebeiPeople’s Republic of China; 2 Department of Biliopancreatic Endoscopic Surgery 71213 The Second Hospital of Hebei Medical UniversityShijiazhuangHebeiPeople’s Republic of China; 3 Biliopancreatic Endoscopic 71213 The Second Hospital of Hebei Medical UniversityShijiazhuangHebeiPeople’s Republic of China

## Case Description



**Video 1** The procedure showing the use of a thin cholangioscope as a rescue option for failed cannulation of altered anatomy bilioenteric strictures via an existing HGS tract.


Bilioenteric strictures after complex surgery are an endoscopic challenge. Cholangioscopy via a hepaticogastrostomy (HGS) tract is an emerging salvage option, particularly with a newer thin cholangioscope.

A 64-year-old woman with a history of Roux-en-Y bilioenteric anastomosis presented with cholangitis revealing intrahepatic bile duct stones and anastomotic strictures, managed with enteroscopy-assisted endoscopic retrograde cholangiopancreatography (EA-ERCP) followed by antegrade endoscopic ultrsound–guided biliary drainage after failed left bile duct cannulation, with placement of a double pigtail stent. She later re-presented with cholangitis, imaging showing recurrent dilation of the left intrahepatic ducts.

EA-ERCP was performed first to clear residual left bile duct stones, exchange the HGS stent, and calibrate the stricture, but cannulation failed. The procedure was converted to cannulation of the intrahepatic ducts through the gastrostomy orifice, followed by the removal of the indwelling stent. Despite multiple attempts, the stricture could not be crossed. A thin cholangioscope (6.5F) was introduced through the HGS orifice, enabling direct visualization and crossing of the stricture under visual control ( Figs. 1 2 3 4 ).

**Fig. 1:**
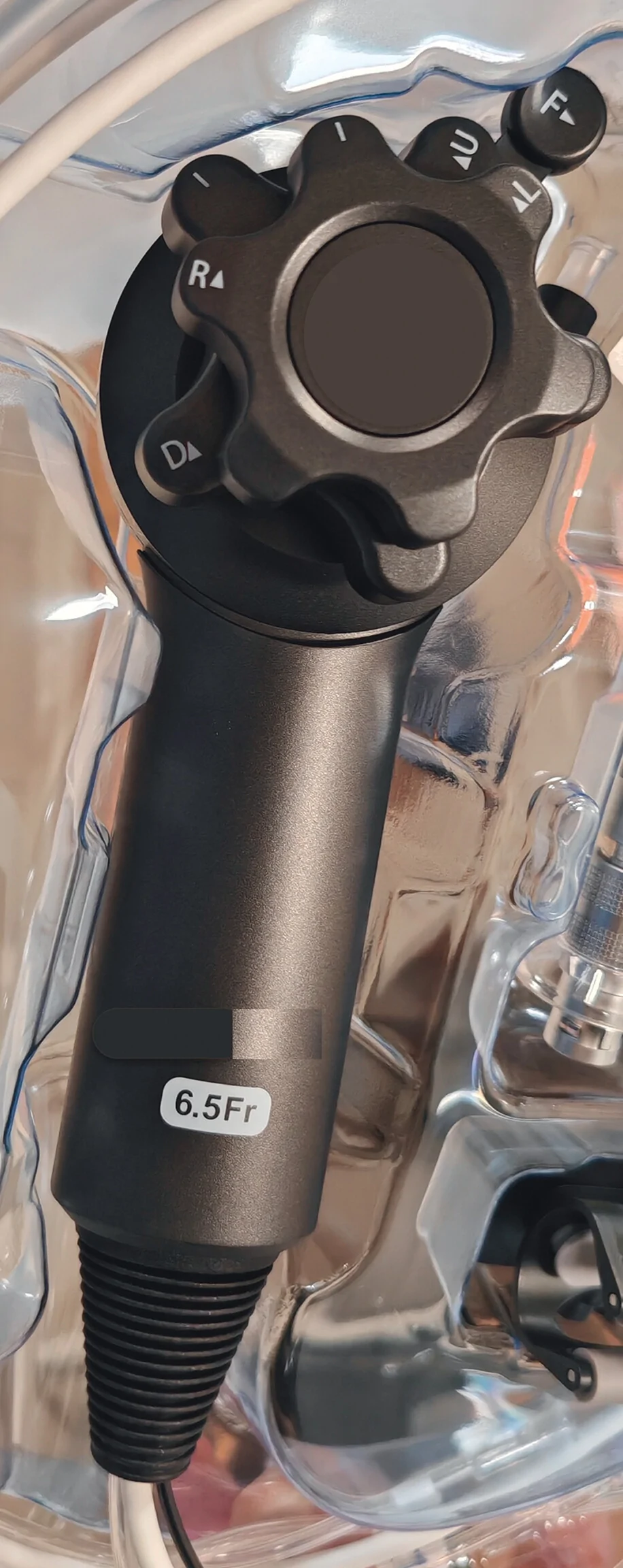
An image showing the thin 6.5F cholangioscope handle.

**Fig. 2:**
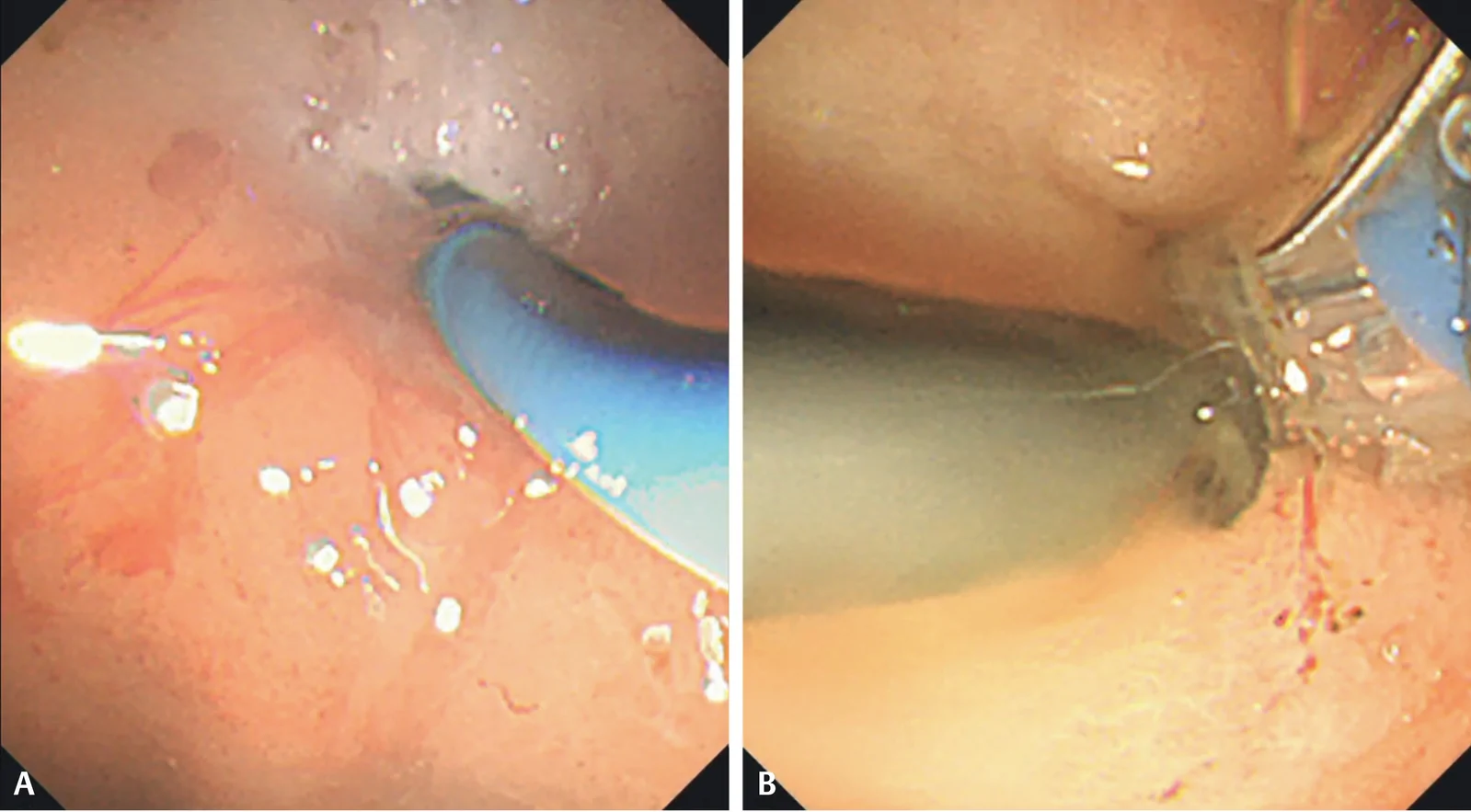
Side by side comparison of the cholangioscope ( **A** ) and the stent ( **B** ) through the HGS tract.

**Fig. 3:**
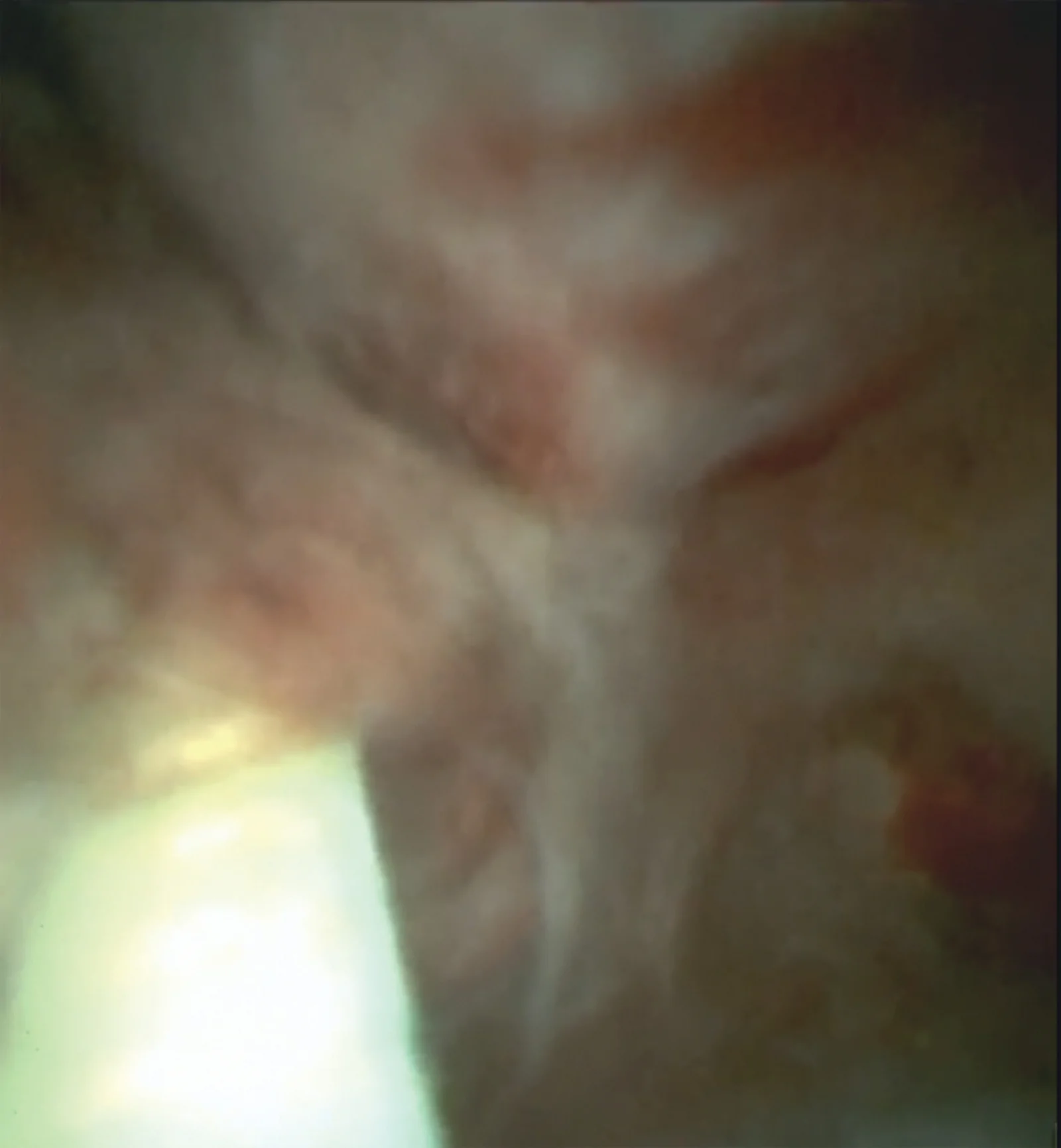
Direct cholangioscopic visualization of the stricture orifice.

**Fig. 4:**
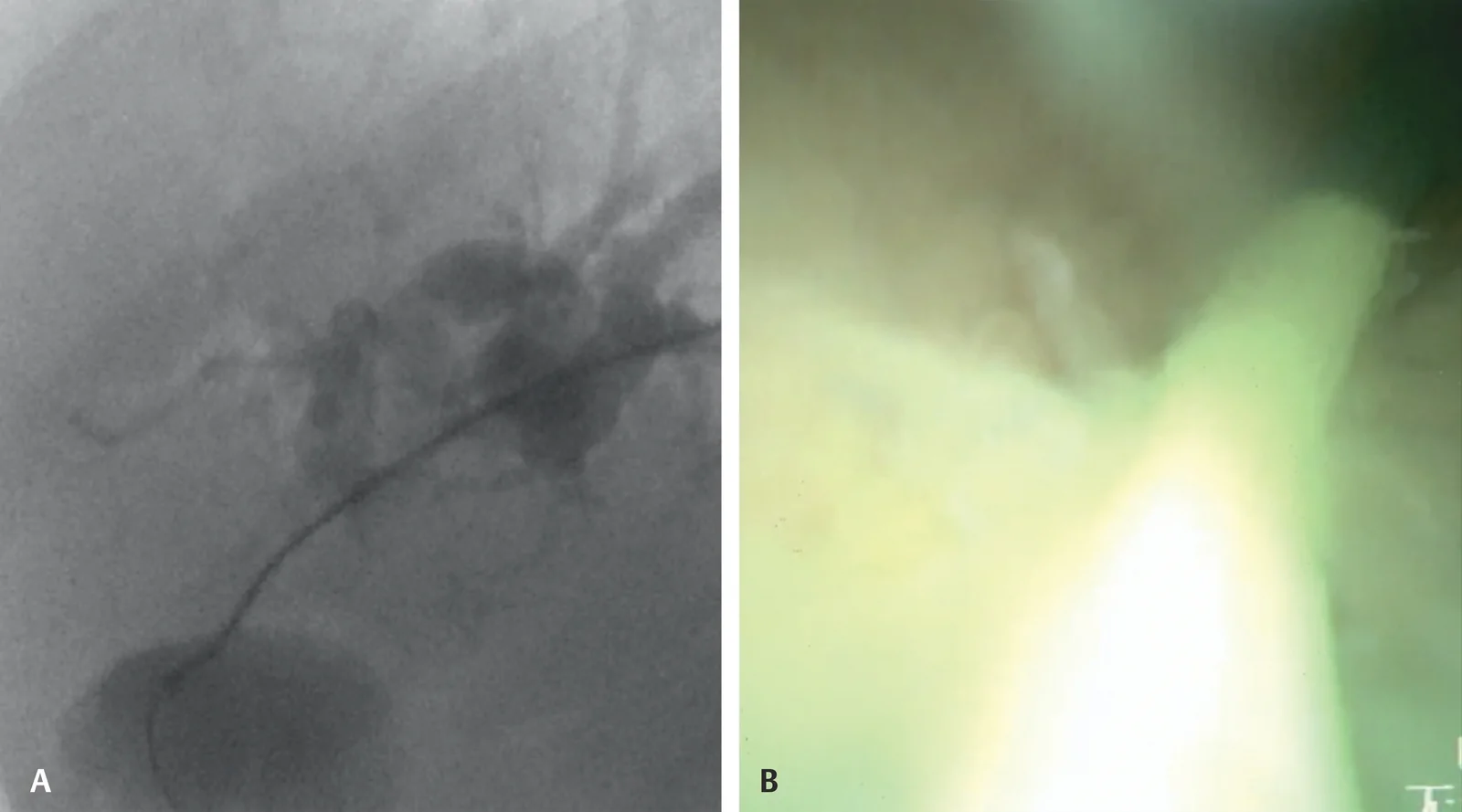
Fluoroscopic ( **A** ) and endoscopic ( **B** ) images confirming the passage into the jejunum.

Bilioenteric strictures after surgical reconstruction are often challenging endoscopic cases. 1​ ​2​ Transluminal cholangioscopy via a percutaneous or HGS tracts offers direct biliary access and visually guided crossing of otherwise unreachable strictures. 3​ ​4​ A 6.5F small-caliber scope facilitates progression through a narrow HGS tract, reducing traumatic risk compared with bulkier devices and allowing for direct visual crossing of a tight stricture or reintervention whether by exchanging or adding stents ( Fig. 5 and Video 1 ).

**Fig. 5:**
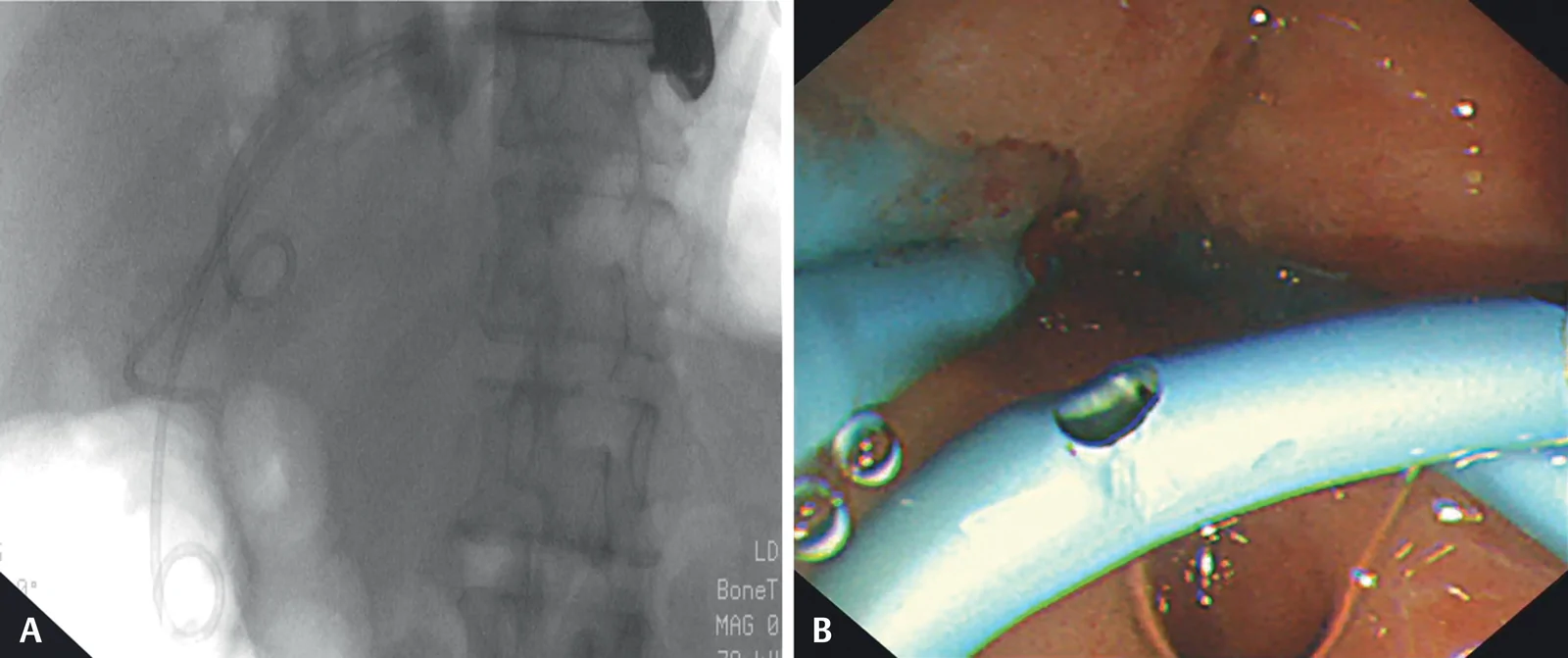
A fluoroscopic ( **A** ) and endoscopic ( **B** ) image showing the three plastic stents placed across the calibrated stricture.

The cholangioscope used is thinner than the plastic stents commonly used in HGS. This demonstrates that a thin cholangioscope can rescue failed cannulation particularly in these settings, 5​ while offering a safe and reproducible alternative to standard cholangioscopes (no further dilation of the tract needed, thus less morbidity and probably higher success odds), and of course to percutaneous or surgical revision.

Endoscopy_UCTN_Code_TTT_1AR_2AG

## Declaration of GenAI Use

The authors confirm that no generative artificial intelligence was used in the preparation of this manuscript.
